# Does Combined Intravenous and Topical Tranexamic Acid Offer Additional Benefit Compared With Intravenous or Topical Alone in Open Elbow Arthrolysis—A Pilot Randomized Controlled Trial

**DOI:** 10.1111/os.70311

**Published:** 2026-04-28

**Authors:** Chen Chen, Jianyu Zhang, Kehan Hua, Weitong Sun, Dan Xiao, Maoqi Gong, Yejun Zha, Xieyuan Jiang

**Affiliations:** ^1^ Department of Orthopedic Trauma, Beijing Jishuitan Hospital Capital Medical University Beijing China; ^2^ Beijing Research Institute of Traumatology and Orthopaedics Beijing China

**Keywords:** blood loss, drainage volume, open elbow arthrolysis, post‐traumatic elbow stiffness, range of motion, tranexamic acid

## Abstract

**Purposes:**

Open elbow arthrolysis (OEA) is a common method for treating post‐traumatic elbow stiffness, which may lead to severe bleeding when resecting heterotopic ossification (HO) and fibrous scar tissue. The purpose of this study is to compare the efficacy and safety among three different ways of using tranexamic acid (TXA) in reducing total blood loss and drainage volume in OEA.

**Patients and Methods:**

This was a pilot, double‐blinded, randomized controlled trial involving a total of 60 patients of post‐traumatic elbow stiffness. Patients in the IV group (*n* = 20) received TXA intravenously, those in the intra‐articular group (*n* = 20) received TXA topically, and those in the combined group (*n* = 20) received TXA both intravenously and topically. The primary outcome measure was postoperative blood loss and drainage volume. The secondary outcome measures included elbow function, complications, and secondary surgery.

**Results:**

The postoperative blood loss on the first and third days and the drainage volume on the first day was similar among the three groups (*p* > 0.05). The total postoperative drainage volume in the IV group was significantly higher than in the combined and intra‐articular group, with no significant difference observed between the combined and intra‐articular group (534.5 mL vs. 378.0 mL vs. 356.5 mL; *p* < 0.05). There were no significant differences in elbow flexion‐extension and rotation range of motion (ROM) and the Visual Analogue Scale (VAS) and Mayo Elbow Performance Score (MEPS) scores at the follow‐up (*p* > 0.05).

**Conclusion:**

Intravenous, topical, or combined TXA administration showed no significant difference in reducing postoperative blood loss after OEA. However, both combined and topical TXA significantly reduced postoperative drainage volume compared to intravenous administration.

**Level of Evidence:**

Therapeutic Level I.

## Graphical abstract

Open elbow arthrolysis is a common method for treating post‐traumatic elbow stiffness. Intravenous, topical, or combined tranexamic acid administration showed no significant difference in reducing postoperative blood loss after open elbow arthrolysis. However, both combined and topical tranexamic acid significantly reduced postoperative drainage volume compared to intravenous administration.

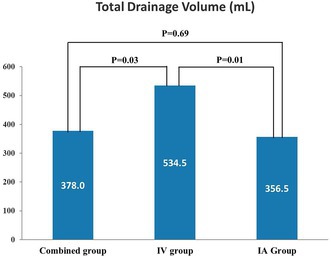

## 1 Introduction

The elbow plays a crucial role in the functionality of the hand. Morrey et al. proposed that the required range of motion (ROM) of the elbow in daily activities includes flexion‐extension (extension of 30° to flexion of 130°) and forearm rotation (50° pronation to 50° supination) [1]. With increased demand for elbow mobility in modern life, limited range of motion leading to impaired daily activities may be diagnosed as elbow stiffness [2]. Elbow stiffness occurs in 10%–15% of patients after elbow trauma [3, 4]. Open elbow arthrolysis (OEA) is a common method for treating post‐traumatic elbow stiffness, as it can restore elbow ROM, thereby improving the quality of life for patients [5, 6].

However, the extensive resection of HO and fibrous scar tissue during OEA can lead to severe bleeding at the surgical site, increasing intraoperative blood loss [7]. Moreover, the trauma caused by the surgery can activate the fibrinolytic system further, leading to an increase in hidden postoperative blood loss. As a result, postoperative drainage volume is significant following OEA, requiring long drainage duration, haematoma, and long hospital stay. Therefore, reducing intraoperative and postoperative blood loss during OEA can decrease the risks associated with perioperative bleeding, facilitating early functional recovery postoperatively.

Tranexamic acid (TXA) is a synthetically derived analogue of lysine that competitively binds to the lysine binding site on plasminogen, preventing fibrinogen binding to that site, thereby inhibiting fibrinolysis and promoting hemostasis [8]. Current studies suggest that tranexamic acid can reduce postoperative blood loss and drainage volume following OEA [7, 9, 10]. However, the optimal route of TXA administration remains unclear. Comparative studies investigating the efficacy and safety of topical, intravenous, or combined TXA application in OEA are still limited. The purposes of this study are as follows: (i) To compare the efficacy of topical, intravenous, and combined administration of TXA in reducing blood loss and drainage after OEA. (ii) To evaluate the safety of the three administration after OEA.

## 2 Materials and Methods

### 2.1 Study Design

This was a pilot, double‐blinded, randomized controlled trial (RCT) to compare different ways of using TXA in reducing blood loss and drainage in patients who had OEA. Approved by the Ethics Committee of Beijing Jishuitan Hospital, Capital Medical University (Approval No. 201904‐10), this study was registered in the Chinese Clinical Trial Registry (ChiCTR1900023124). Written informed consent was obtained from all patients.

### 2.2 Patients

Patients who underwent OEA in Beijing Jishuitan Hospital from June 2020 to September 2021 were eligible for this study. All the patients included had post‐traumatic elbow stiffness with HO and were treated with OEA by two surgeons. The exclusion criteria include: allergy to TXA; deep vein thrombosis or pulmonary embolism history; history of coagulation dysfunction; hepatic or renal disorder; on anticoagulation or hormone replacement management.

### 2.3 Randomization, Blinding and Interventions

After enrollment, the patients were randomized into three groups based on a computer‐generated list with a block size of 6. Research assistant sealed the allocation sequence number in an opaque envelope until the morning of surgery. Prior to the surgery, the envelope was opened by the research assistant with the nurse and anesthetist together who prepared the TXA methods and for safety. Patients, surgeons, all the care providers, and outcome analysts were blinded to the randomization. Each of the two surgeons performed 10 procedures in every group, thereby minimizing operator‐related bias.

Patients were randomized to three groups: IV, intra‐articular and combined group. TXA intra‐articular group received 100 mL saline solution intravenously and 1 g of TXA diluted in 10 mL saline solution which was injected in the joint. TXA IV group received 15 mg/kg TXA with 100 mL saline solution administered intravenously and 10 mL of saline injected intra‐articularly. Combined group received combined administration of TXA: 15 mg/kg of TXA diluted in 100 mL saline solution (0.9%), administered intravenously 10 min before skin incision. 1 g of TXA diluted in 10 mL of saline solution TXA was injected anteriorly and posteriorly in the elbow joint evenly with needle after closure of skin, drainage was kept closed for 2 h.

### 2.4 Surgery and Postoperative Care

All OEA were performed by two authors (C.C and Z.YJ.) who were experienced elbow surgeons. All patients underwent a brachial plexus blocking anesthesia. OEA used a medial‐lateral combined approach, following a basic sequence which contained ulnar nerve decompression and posterior capsule excision through the medial approach, anterior capsule excision through the lateral approach. All blocking HO were excised and contracted tissue was removed to reach the intra‐operation passive ROM of 0°–130° as sufficient. After meticulous electrocoagulation, 2 drainages were placed in the anterior and posterior part of the joint through the lateral and medial approach separately. Fascia and skin were closed layer by layer. Drainage was clamped for the first 2 h post‐op to avoid TXA loss from the joint. After closure, varus/valgus stability was tested and no external fixator was used due to careful preservation of important ligaments found. Patients started rehabilitation on the first day after surgery with the help of our experienced physiotherapist.

Patients were encouraged to leave the bed immediately after surgery for venous thromboembolism prophylaxis with pain control. The criteria for removing the drainage tube were as follows: starting from the third postoperative day, the daily drainage volume per tube was less than 30 mL, or the daily drainage volume per tube was less than 50 mL for three consecutive days. All patients were discharged after the removal of their drainage tubes. Transfusion protocol was based on guidelines of the Chinese Ministry of Health; patients with Hb < 70 g/L or symptomatic patients with Hb 70–100 g/L were planned for transfusion.

### 2.5 Outcome Measures

The primary outcome measure was total blood loss of the first day and third day post‐operation and drainage volume. The secondary outcome measures were elbow function. The total blood loss was calculated with Gross formula [11] using the difference of hemoglobin. Estimated total blood volume was calculated according to Nadler's formula [12]. The same author (C.C) performed the measurement of blood loss. Transfusion was recorded. Patients were scheduled to go back to outpatient at 1, 3, 6, and 12 months for follow‐up and rehabilitation. Elbow function included Visual Analogue Scale (VAS) for pain, ROM, and Mayo Elbow Performance Score (MEPS). Complications and secondary surgery were recorded at each follow‐up including thromboembolic events, infection, hematoma.

### 2.6 Sample Size and Statistics Analysis

PASS (Power Analysis and Sample Size) 2021 Statistical Software (Utah, USA) was used to estimate the sample size. PASS 2021 software provided Pilot Study Sample Size Rules of Thumb for finding an appropriate sample size for a pilot study in which the outcome was a continuous measurement (postoperative blood loss in our study). The procedure was based on the flat rules of thumb, provided by Machin et al. [13] (Figure 2). These rules of thumb for two groups could be adapted for single‐group or multi‐group studies by multiplying the recommended pilot study sample size by an appropriate adjustment factor (e.g., 0.5 for a study with only one group or 1.5 for a study with three groups, etc.). The results showed the sample size should be no less than 10 for a study with only one group, no less than 20 for a study with two groups or no less than 30 for a study with three groups [13, 14, 15, 16, 17]. A total of 60 patients, 20 in each group, were included in our study.

Quantitative data were presented as mean and SD or median with the interquartile range (IQR) based on if it was normally distributed which were assessed by Shapiro–wilk test. Qualitative data were presented as frequencies and percentages. The continuous variables were compared using one‐way analysis of variance or non‐parametric test and the categorical variables were compared using the chi‐square test. All data were analyzed using SPSS software (version 24.0; IBM), *p* value of < 0.05 was considered significant.

## 3 Results

### 3.1 Study Population

**FIGURE 1:**
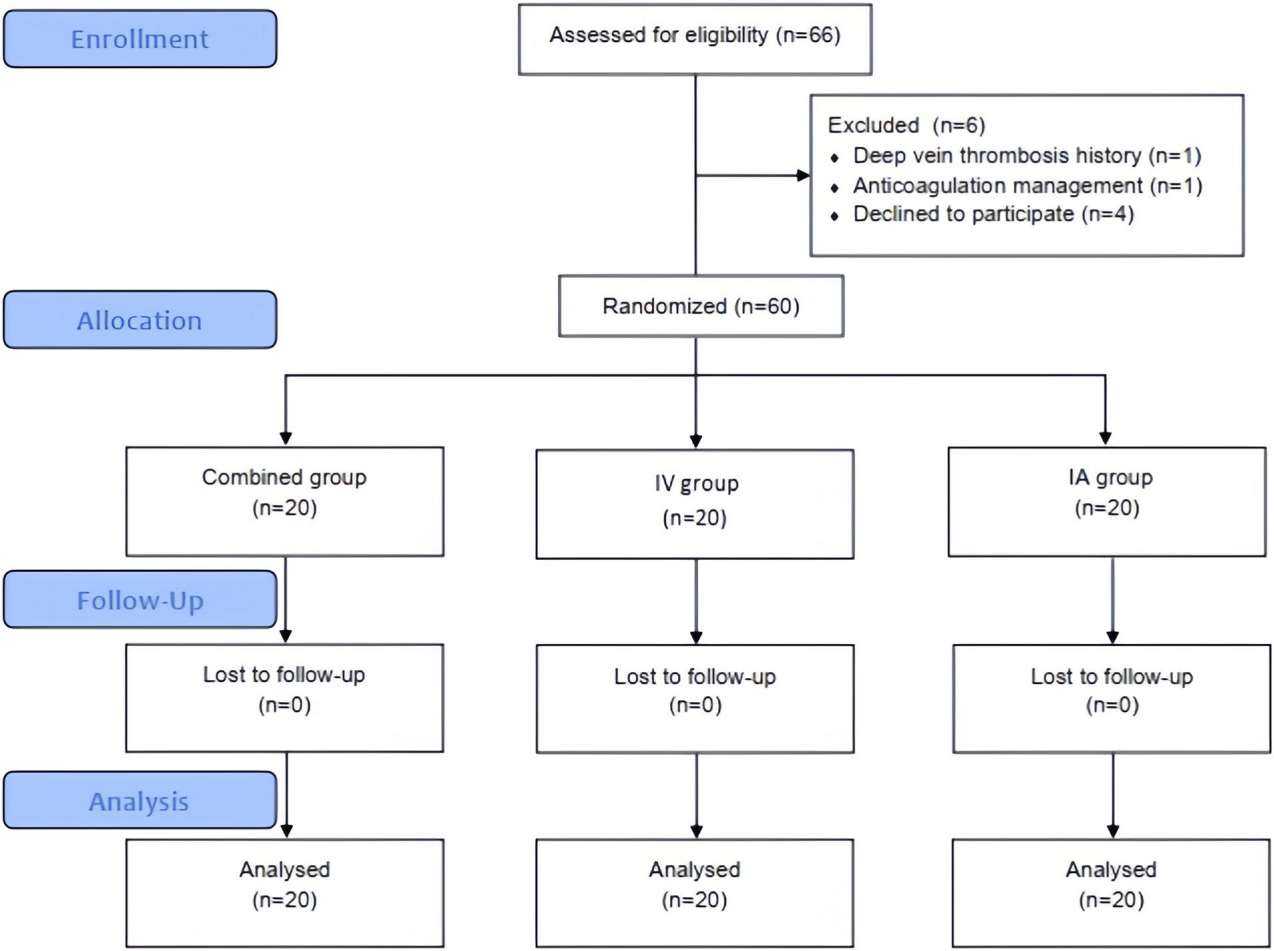
Flow of patients for the study.

66 patients met the inclusion criteria in the study, but 2 patients were ineligible according to the exclusion criteria and 4 patients refused. Ultimately, a total of 60 patients were enrolled in this study, and the combined group, IV group, and intra‐articular group each included 20 patients (Figure 1). All patients included in the study were followed for at least 12 months. The baseline and preoperative values of the three groups showed no significant differences (*p* > 0.05, Table 1).

**TABLE 1:** Patients' baseline characteristics of the study.

	Combined group (*N* = 20)	Single IV group (*N* = 20)	Single intra‐articular group (*N* = 20)	F/χ^2^	*p*
Age (*year*)^a^	36.4 (12.1)	39.2 (10.8)	36.3 (12.6)	0.38	0.69
Gender (*no. [%]*)				1.6	0.45
Male	10 (50.0%)	12 (60.0%)	8 (40.0%)		
Female	10 (50.0%)	8 (40.0%)	12 (60.0%)		
Weight (*kg*)^a^	65.6 (14.5)	71.0 (12.0)	63.7 (11.6)	1.73	0.19
Height (*m*)^a^	1.7 (0.1)	1.7 (0.1)	1.7 (0.1)	0.72	0.49
BMI(*kg/m* ^*2*^)^a^	23.4 (4.5)	24.4 (3.9)	22.2 (2.9)	1.64	0.20
Preoperative values^a^					
Hemoglobin (*g/dL*)	145.1 (16.7)	142.1 (19.1)	143.2 (12.3)	0.18	0.84
Hematocrit (*%*)	42.7 (4.2)	42.2 (4.9)	42.6 (3.6)	0.08	0.93
Platelet count (*×10* ^*9*^ */L*)	233.5 (54.5)	234.0 (53.0)	241.8 (51.3)	0.15	0.86
Blood Volume (*L*)	4.3 (0.87)	4.6 (0.70)	4.2 (0.8)	1.30	0.28
Preop. elbow function^a^					
Flexion‐extension ROM (*deg*)	31.6 (32.3)	24.7 (30.6)	46.7 (26.4)	2.84	0.09
Rotation ROM (*deg*)	132.6 (50.2)	130.2 (33.8)	131.3 (55.5)	2.40	0.33
VAS	2.3 (2.5)	1.95 (2.4)	2.4 (2.1)	0.20	0.63
MEPS	59.3 (14.9)	56.5 (14.0)	59.3 (12.0)	0.27	0.76

^a^ The values are given as the mean and standard deviation.

### 3.2 Postoperative Blood Loss and Drainage

**FIGURE 2:**
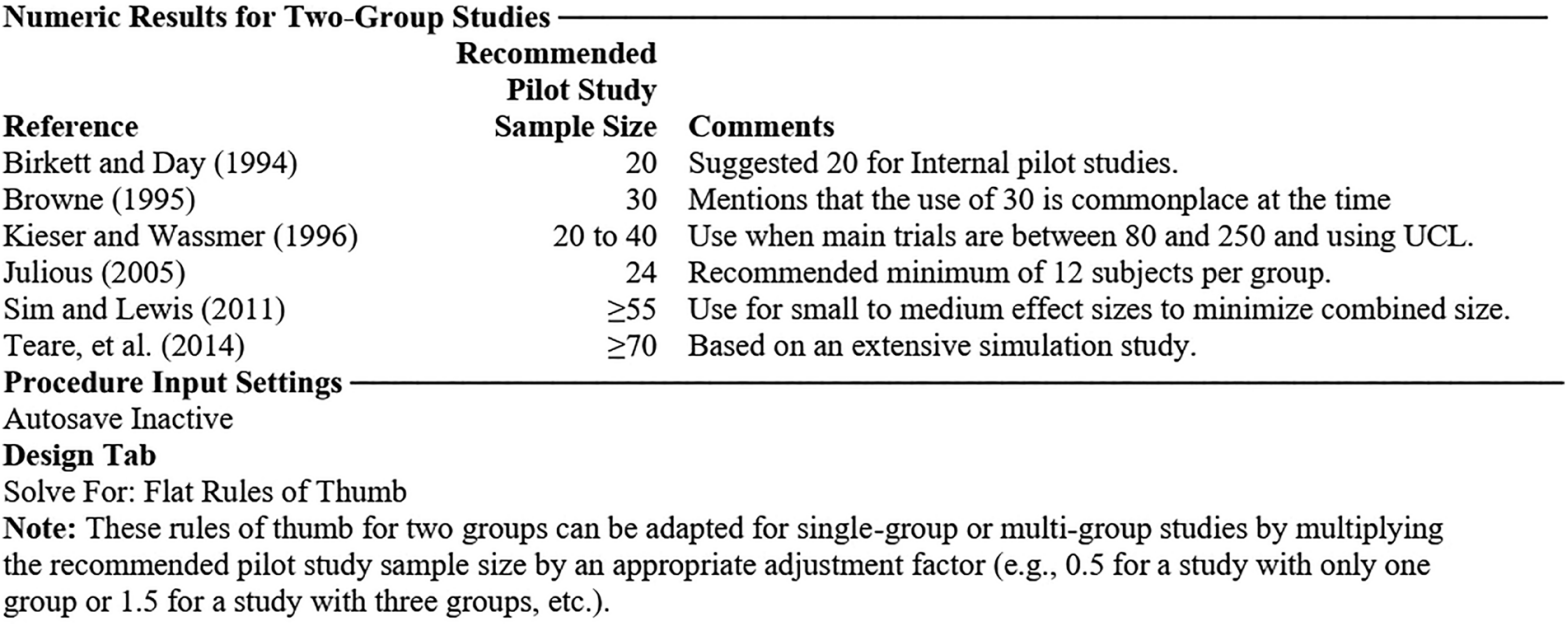
Results of pilot study sample size rules of thumb.

**FIGURE 3:**
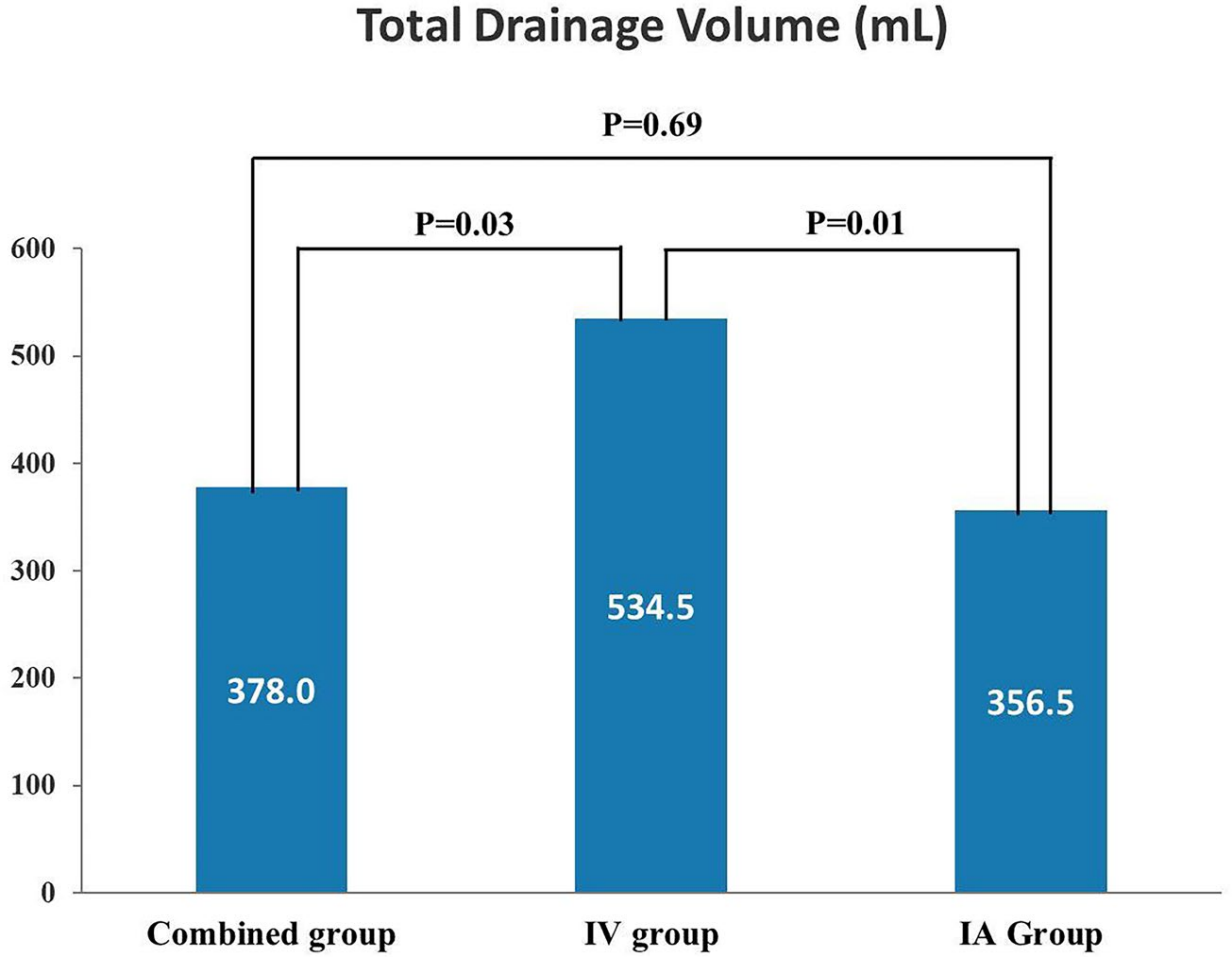
The mean total drainage volume and the intergroup differences of the three groups.

Duration of postoperative hospital stay and the postoperative blood loss on the first and third days was comparable among the three groups, with no statistically significant differences (*p* > 0.05, Table 2). Similarly, the drainage volume on the first day postoperative showed no significant differences among the three groups (*p* > 0.05). However, the total drainage volume in the combined and intra‐articular group was significantly lower than in the IV group, with no significant difference observed between the combined and intra‐articular group (378.0 mL vs. 356.5 mL vs. 534.5 mL; *p* < 0.05, Figure 2). Additionally, the drainage time was similar across all groups (Figure 3).

**TABLE 2:** Patient' primary and secondary outcomes.

	Combined group (*N* = 20)	Single IV group (*N* = 20)	Single intra‐articular group (*N* = 20)	F	*p*
Duration of postoperative hospital stay (*day*)^a^	5.4 ± 1.2 (4.7, 6.0)	5.5 ± 1.5 (4.7, 6.2)	5.3 ± 1.3 (4.8, 5.9)	0.03	0.94
Blood loss on 1st postop. day (*mL*)^a^	522.7 ± 319.6 (373.1, 672.3)	578.8 ± 451.4 (367.5, 790.0)	565.2 ± 265.3 (441.0, 689.4)	0.04	0.87
Blood loss on 3rd postop. day (*mL*)^a^	770.6 ± 301.8 (629.4, 911.9)	904.9 ± 456.3 (691.3, 1118.4)	846.7 ± 404.2 (657.5, 1035.9)	0.38	0.56
Drainage on 1st postop. day (*mL*)^a^	165.5 ± 128.0 (105.6, 225.4)	248.0 ± 191.9 (158.2, 337.8)	128.5 ± 78.3 (91.9, 165.1)	3.79	0.12
Postop. total drainage (*mL*)^a^	378.0 ± 175.4 (295.9, 460.1)	534.5 ± 261.3 (412.2, 656.8)	356.5 ± 160.5 (281.4, 656.8)	4.54	**0.02**
Drainage duration (*day*)^a^	3.9 ± 0.9 (3.8, 4.3)	4.7 ± 1.7 (3.9, 5.4)	4.3 ± 1.0 (3.8, 4.7)	1.87	0.35
Postop. elbow function^a^					
Flexion‐extension ROM (*deg*)	125.9 ± 10.6 (120.9, 130.8)	126.0 ± 11.9 (120.4, 131.6)	126.8 ± 11.1 (121.6, 132.0)	0.05	0.96
Rotation ROM (*deg*)	157.4 ± 21.7 (147.2, 167.5)	156.3 ± 20.9 (146.5, 166.1)	162.8 ± 21.0 (153.0, 172.6)	0.55	0.26
VAS	0.5 ± 1.1 (0, 1.0)	0.4 ± 0.8 (0, 0.7)	0.4 ± 0.8 (0, 0.8)	0.15	0.83
MEPS	96.8 ± 6.1 (93.9, 99.6)	94.8 ± 8.5 (90.8, 98.7)	95.3 ± 7.5 (91.7, 98.8)	0.39	0.70

*Note:* Bold indicates statistical significance.

^a^ The values are given as the mean, standard deviation, and 95% confidence interval.

### 3.3 1‐Year Follow‐Up Outcomes

At the one‐year follow‐up, there were no significant differences in elbow flexion‐extension and rotation ROM and the VAS and MEPS scores among the three groups (*p* > 0.05, Table 2). No blood transfusions were required in any of the groups, and no hemorrhage and coagulation‐related complications such as hematoma, deep vein thrombosis, or infection were observed. Moreover, no secondary surgeries were performed.

## 4 Discussion

### 4.1 Key Findings and Clinical Implications

The findings of this study investigating intravenous, topical, and combined TXA administration in OEA are as follows: no significant differences were observed in blood loss at 1 and 3 days postoperatively, 1‐year elbow functional outcomes. Notably, topical and combined TXA administration both achieved a significant reduction in total postoperative drainage volume compared with intravenous TXA administration, while no significant difference was detected between the topical and combined groups.

Currently, there are some studies regarding the impact of TXA on OEA. Despite concerns about the potential for intravenous TXA to cause deep vein thrombosis or pulmonary embolism, a systematic review and meta‐analysis indicated that intravenous TXA reduces drainage volume without affecting elbow ROM or complications rate [18]. Although there are few studies on the topical application of TXA in OEA, extensive research in knee and hip arthroplasty has confirmed its hemostatic efficacy and safety [19, 20, 21]. The study of Zhang et al. demonstrated that the topical application of 1 g TXA significantly reduces intraoperative blood loss and drainage volume in OEA [9]. These studies have demonstrated that the use of TXA in OEA can reduce bleeding without increasing the complication rate. These existing studies have solidly established that TXA administration in different routes can reduce bleeding‐related outcomes in OEA without elevating the incidence of complications, which also provides the important clinical rationale for our study to not set a blank control group and further compare the efficacy of different TXA administration.

Despite the combined group having less blood loss compared to the other two groups, there was no statistically significant difference on the first and third postoperative days. This finding is supported by the prior literature. A study on bilateral total knee arthroplasty also suggested that combined and topical administration of TXA did not show a significant difference in blood loss [22]. The total drainage volume in the combined and intra‐articular groups was similar, both being less than that in the IV group (*p* < 0.05), suggesting that these two methods may have potential advantages. Drainage tubes were routinely placed postoperatively in all patients to mitigate the risk of local hematoma formation. All patients followed the same criteria for drain removal. Although there was no statistically significant difference in drainage duration among the three groups, the combined group had the shortest duration, followed by the intra‐articular group. Reduced drainage volume and earlier drain removal facilitate free early mobilization, which is beneficial for restoring elbow ROM. Furthermore, the reduction in drainage volume contributed to a decreased nursing burden and improved patient comfort. Although no statistically significant difference in drainage duration was observed among the three groups, it is noteworthy that the IV group exhibited the longest duration.

### 4.2 Safety and Dosage Considerations

Regarding safety, our findings are consistent with previous studies [7, 9, 10]. In this study, none of the patients experienced complications such as hematoma, deep vein thrombosis, or pulmonary embolism. Since postoperative patients were not bedridden, the risk of lower extremity deep vein thrombosis was low. Adequate drainage also reduced the risk of hematoma. Therefore, the use of TXA in OEA is relatively safe.

Dosage selection and administration approach for TXA in this study were determined based on the anatomical characteristics of the elbow and evidence from existing clinical research. Based on previous studies in total knee arthroplasty, the maximum TXA dose is typically around 3 g in total knee arthroplasty studies [23]. However, the elbow joint has less capacity. Currently, researchers commonly use 1 g TXA administered intravenously or topically in OEA [7, 9, 10, 24]. Compared to previous studies where TXA was soaked in gauze, we chose to inject TXA directly into the elbow joint cavity to increase the drug concentration. In this study, we administered TXA intravenously at a dose of 15 mg/kg and topically at a dose of 1 g. Our chosen dosages were deemed safe, and the results confirm this [25]. The combined application of TXA in our study represents a novel approach.

### 4.3 Strengths and Limitations

This research is the first pilot randomized controlled trial to compare the efficacy and safety of intravenous, topical, and combined TXA administration in OEA, filling the research gap for this surgical procedure. And it adopted a rigorous design with standardized surgical and postoperative rehabilitation protocols, which minimized operator‐related and selection biases and ensured the internal validity of the research findings. This study has several limitations. First, as a pilot study, it included a relatively small number of patients; a larger sample size might better reveal intergroup differences and lead to more definitive conclusions. Second, the ethical considerations limited the practicality of a blank control group who would receive no TXA. Third, the dosage design for different groups in this study was singular, and future research could investigate varying dosages. Lastly, this study was conducted at a single center, which limits the generalizability of the findings. Future research should include multicenter studies adhering to the same standards.

## 5 Conclusion

The conclusion of this study is that intravenous, topical, or combined administration of TXA shows no significant differences in postoperative blood loss, elbow function, or complications following OEA. However, compared to intravenous administration, both combined and topical administration of TXA significantly reduce postoperative drainage volume, which may offer potential advantages.

## Author Contributions

**Jianyu Zhang:** writing – original draft, writing – review and editing, data curation, formal analysis, visualization. **Chen Chen:** conceptualization, methodology, data curation, writing – review and editing, writing – original draft, formal analysis, visualization. **Kehan Hua:** conceptualization, validation, methodology, data curation. **Weitong Sun:** formal analysis, visualization, methodology, supervision. **Yejun Zha:** resources, project administration, formal analysis, supervision, visualization, writing – original draft. **Maoqi Gong:** project administration, supervision. **Dan Xiao:** visualization, formal analysis, conceptualization, methodology, supervision. **Xieyuan Jiang:** supervision, funding acquisition, visualization, project administration, and resources.

## Funding

This research was supported by Young Elite Scientists Sponsorship Program by Beijing Association for Science and Technology (No. BYESS2023115), Beijing Natural Science Foundation (L244014), and National Key Research and Development Program of China (2024YFC3044700).

## Ethics Statement

The study was approved by Beijing Jishuitan Hospital Ethics Board and this study was registered in the Chinese Clinical Trial Registry (ChiCTR1900023124).

## Conflicts of Interest

The authors declare no conflicts of interest.

## Data Availability

The data that support the findings of this study are available on request from the corresponding author. The data are not publicly available due to privacy or ethical restrictions.
